# Qualitative exploration of women’s experiences of vasomotor symptoms to support the content validity of patient-reported outcomes

**DOI:** 10.1186/s41687-025-00914-0

**Published:** 2025-07-01

**Authors:** Claudia Haberland, Melissa Barclay, Sophie Whyman, Asha Lehane, Adam Gater, Christoph Gerlinger, Christian Seitz, Maja Francuski, Nils Schoof, Andrew Trigg, Helena Bradley

**Affiliations:** 1 Bayer AG, Berlin, Germany; 2 Adelphi Values, Cheshire, UK; 3 Department of Gynecology, Obstetrics and Reproductive Medicine, University Medical School of Saarland, Homburg/Saar, Germany; 4 Institute of Clinical Pharmacology and Toxicology, Charité – Universitätsmedizin Berlin, Berlin, Germany; 5 Medical Affairs Statistics, Bayer plc, Reading, UK

**Keywords:** Vasomotor symptoms, Hot flashes, Menopause, Patient-reported outcome, Conceptual model, Content validity, Meaningful change

## Abstract

**Background:**

Frequency and severity of vasomotor symptoms (VMS; hot flashes) associated with menopause significantly impact women’s health-related quality of life (HRQoL). Treatment benefit in VMS clinical trials is assessed using patient-reported outcome (PRO) measures, which must demonstrate evidence of content validity. This research aimed to establish a conceptual model in VMS and evaluate content validity of the Hot Flash Daily Diary (HFDD), PROMIS Sleep Disturbance Short Form 8b (PROMIS SD SF 8b), and Menopause-Specific Quality of Life (MENQOL) questionnaire for use in VMS clinical trials.

**Methods:**

Targeted searches were conducted to identify qualitative literature documenting women’s VMS experiences. Qualitative concept elicitation (CE) and cognitive interviews (CI) were then conducted with 20 US participants experiencing VMS (*n* = 10 postmenopause; *n* = 10 AET-treated). Literature and CE findings were used to develop a conceptual model and confirm conceptual coverage of PRO measures selected for assessing efficacy in VMS clinical trials. CIs assessed the content validity of PRO measures.

**Results:**

Findings from the literature and CE interviews informed a conceptual model depicting women’s experiences of VMS. Thirty-three symptom concepts were identified with key symptoms including sweating, cold sweats/chills and tiredness/fatigue. Impacts of VMS on HRQoL were categorized into seven domains: sleep, emotional wellbeing, activities of daily living, social wellbeing, work/education, cognitive and physical functioning. The concepts assessed by the HFDD (VMS severity and frequency), PROMIS SD SF 8b (sleep disturbance) and MENQOL (menopause-related quality of life) aligned with those endorsed by women as relevant to their VMS experience. Instructions, recall periods and response options of the measures were understood. A reduction of one moderate or one severe hot flash in 24-hours (assessed by the HFDD) was considered a meaningful improvement by participants. Similar observations were made across study samples.

**Conclusions:**

Findings provide detailed insights into women’s experience of VMS, supporting the development of a conceptual model and assessment of conceptual coverage of selected PRO measures. Content validity of the HFDD, PROMIS SD SF 8b, and MENQOL for use in VMS clinical trials was supported. CI results suggest that a reduction of one moderate or one severe hot flash in 24-hours is meaningful to women with VMS.

**Supplementary Information:**

The online version contains supplementary material available at 10.1186/s41687-025-00914-0.

## Background

The menopausal transition reflects the natural decline of follicular estrogen production as a consequence of ovarian aging. Physiologically, this transition stretches over several years (perimenopause), characterized by increased variability in sex hormone levels and menstruation. This results in the post-menopausal state, with low estradiol levels and permanent absence of menstruation. Menopause can also be induced by medical intervention (e.g., surgical removal of both ovaries or adjuvant endocrine therapy [AET]), resulting in a sudden drop of estrogen levels (induced menopause) [1].

Vasomotor symptoms (VMS) and sleep disturbances are some of the most commonly reported and bothersome symptoms associated with menopause [2] and pose substantial impacts on women’s health-related quality of life (HRQoL) [3–7]. VMS are reported by up to 80% of women during the menopausal transition and last for a median duration of 7.4 years [8]. Based on a cross-cultural survey of postmenopausal women, more than 60% reported difficulty sleeping and over 50% reported feeling anxious/nervous and/or depressed [2]. Consequently, VMS are a leading cause for seeking medical attention and there is a growing focus on the development of pharmacological and non-pharmacological therapies for the treatment of VMS [9].

Hormone therapy remains the most effective option for the management of menopause-related VMS [10]. However, many women cannot use hormonal therapies due to medical contraindications or choose not to use them [11–13]. Elinzanetant is a non-hormonal, selective neurokinin 1 and 3 (NK-1, 3) receptor antagonist in development for the treatment of moderate-to-severe VMS. Preclinical and clinical data indicate these receptors are involved in pathways that trigger the occurrence of VMS in a situation of estrogen depletion [14]. Inhibition of the NK-3 receptor results in improvement in VMS in postmenopausal women [14]; specific inhibition of the NK-1 receptor has been shown to improve primary insomnia [15]. Elinzanetant resulted in significant improvements in VMS, sleep disturbances and menopausal aspects of HRQoL and was well tolerated in a Phase 2 and in Phase 3 clinical trials enrolling postmenopausal women experiencing moderate-to-severe VMS [16, 17].

VMS treatment effect can either be measured subjectively (e.g., patient-reported outcome (PRO) measures) or objectively (e.g., skin conductance levels). While objective measures provide valuable insights, studies have demonstrated discordance with self-reported measures of hot flashes [18]. Self-reported measures are recommended by regulatory agencies to ensure women’s experiences of hot flashes are captured and meaningfully incorporated into drug development [19–22]. Regulatory guidance requires that self-reported changes in the frequency and severity of moderate-to-severe VMS (according to clinical definitions based on women’s experience and report of heat, sweating and impact on activities) are used to assess primary efficacy endpoints in VMS clinical trials [21, 23]; information that has typically been captured via patient-reported diaries [24, 25]. However, FDA requirements relating to assessment of VMS pre-date current regulatory guidance to support product labelling. For PROs to be considered fit-for-purpose, current guidance emphasizes the need to demonstrate evidence of content validity and measurement properties (e.g., reliability, validity, ability to detect change, interpretation of scores) within the context of use [19–21, 26].

The Hot Flash Daily Diary (HFDD) was developed to assess treatment efficacy on the frequency and severity of moderate-to-severe hot flashes. Although developed in line with regulatory guidance, evidence of content validity in the target population has not yet been formally established. The Patient-Reported Outcomes Measurement Information System Sleep Disturbance Short Form 8b (PROMIS SD SF 8b) was identified as an appropriate measure for assessing sleep disturbances in VMS [27]. While a recent study provided evidence of content validity of the PROMIS SD SF 8b in VMS associated with menopause [28], no evidence currently exists for its content validity in VMS associated with AET. Menopausal aspects of HRQoL are also of interest in VMS. The Menopause-Specific Quality of Life (MENQOL) questionnaire was developed to assess HRQoL in menopausal women [29] and is frequently used in VMS clinical trials [25], with evidence of content validity identified in early postmenopausal women [29], but not specifically in postmenopausal or AET-treated women experiencing VMS.

The objective of this qualitative research was to explore the experience of VMS in postmenopausal and AET-treated women and evaluate content validity of the HFDD, PROMIS SD SF 8b and MENQOL to support their inclusion for assessing clinical efficacy endpoints in pivotal VMS clinical trials.

## Methods

This qualitative research comprised a targeted qualitative literature review and combined concept elicitation (CE) and cognitive interviews (CI; also known as cognitive debriefing) with women experiencing VMS from the United States (US). A conceptual model depicting the experience of VMS was developed and content validity of the HFDD, PROMIS SD SF 8b, and MENQOL, for assessing efficacy in VMS clinical trials, was evaluated. Concepts within the model were mapped to these PROs to evaluate their conceptual comprehensiveness (the extent key concepts of interest are captured). Table 1 provides an overview of the PROs. Patient global impression of severity (PGI-S) and change (PGI-C) items were also debriefed to support their use as anchors in future quantitative analyses [22].

**Table 1:** Overview of PRO measures assessed as part of the study

PRO measure	Description
Hot Flash Daily Diary (HFDD)	The HFDD is a diary completed twice daily (morning and evening) to assess the number of mild (sensation of heat without sweating), moderate (sensation of heat with sweating, but able to continue activity), and severe (sensation of heat with sweating, causing cessation of activity) hot flashes experienced during the night and during the day, the number of night-time awakenings and the severity of sleep disturbances due to hot flashes at night. The HFDD utilizes three response types: (1) dichotomous ‘yes’ or ‘no’ for items assessing whether hot flashes are experienced during the day or night; (2) scroller fields to capture responses to items assessing the number of hot flashes experienced of differing severities and the number of night-time awakenings for the Morning HFDD; and (3) a 5-point verbal rating scale (VRS) from 1 (‘not at all’) to 5 (‘very much’) for the HFDD items measuring sleep disturbance due to hot flashes.
Patient-Reported Outcomes Measurement Information System Sleep Disturbance Short Form 8b (PROMIS SD SF 8b) [27]	The PROMIS SD SF 8b is an 8-item generic measure designed to assess self-reported perceptions of sleep disturbances including: sleep quality and restoration with sleep, perceived difficulties or concerns about getting to sleep or staying asleep and adequacy of and satisfaction with sleep over the past seven days. Three different 5-point VRSs are used: the items on restless sleep, satisfaction with sleep, refreshing sleep and difficulty falling asleep use a VRS of ‘not at all’, ‘a little bit’, ‘somewhat’, ‘quite a bit’ until ‘very much’; items assessing trouble staying asleep, trouble sleeping or getting enough sleep use VRS of ‘never’, ‘rarely’, ‘sometimes’, ‘often’ and ‘always’. Sleep quality is measured on a 5-point VRS from ‘very poor’, to ‘very good’.
Menopause-Specific Quality of Life Questionnaire (MENQOL) [29]	The MENQOL is comprised of 29 items across four domains of symptoms and functioning: VMS (also referred to as Vasomotor domain), Psychosocial functioning, Physical functioning, and Sexual functioning. For each item, the participant indicates if they have experienced the problem in the past week (yes/no). If the participant selects ‘yes’, they then rate how bothered they were by the problem using a seven-point numeric rating scale, with response options ranging from 0 ‘not at all bothered’ to 6 ‘extremely bothered’. If the participant selects ‘no’, then no further rating is required for the given item.
Patient-Global Impression of Severity (PGI-S) items	Three single-item PGI-S measures were developed to correspond with the concepts assessed by the HFDD: frequency and severity of hot flashes, and severity of sleep disturbances. Each PGI-S asks participants to choose the response that best describes how often, or severe their hot flashes/sleep disturbances have been over the past week. A 5-point VRS is used for the hot flash frequency PGI-S item (‘no hot flashes’, ‘rarely’, ‘sometimes’, ‘often’, ‘very often’) and for the hot flash and sleep disturbance severity PGI-S items (‘no hot flashes/sleep disturbances’, ‘mild, ‘moderate, ‘severe, ‘very severe’).
Patient-Global Impression of Change (PGI-C) items	Three single-item PGI-C measures were developed to correspond with the concepts assessed by the HFDD. Each PGI-C asks participants to choose the response that best describes the overall change in the frequency or severity of hot flashes/sleep disturbances since taking the study medication. A 5-point VRS is used for the hot flash frequency PGI-C item (‘much less’, ‘a little less’, ‘the same [no change]’, ‘a little more’, ‘much more’ and for the hot flash and sleep disturbance severity PGI-C items (‘much better’ ‘a little better’, ‘the same [no change]’, ‘a little worse’, ‘much worse’).

### Targeted qualitative literature review

For the targeted review of published qualitative literature, searches were conducted in September 2020 and 2023 via bibliographic databases including MEDLINE, Embase and PsycINFO, using a combination of keywords and Medical Subject Headings (Supplementary Tables 1 and 2). Supplementary searches of Google Scholar and key conferences were also performed. Abstracts identified were screened and graded according to the extent they met the study objectives by two researchers trained and experienced in conducting qualitative research (Supplementary Tables 3–6). Abstracts which focused on the qualitative experience of VMS in postmenopausal or AET-treated women and referenced a concept of interest (e.g., hot flash) were prioritized for review.

Full-text articles were reviewed and relevant signs/symptoms and HRQoL impacts relating to women’s experiences of VMS were extracted for analysis. For articles describing the experience of AET-treated women, symptoms or impacts reported to be related to another treatment (e.g., chemotherapy) or the experience of cancer itself (e.g., recurrence fears) were excluded. Findings were used to develop a draft conceptual model displaying the key concepts associated with VMS.

### Qualitative interviews

#### Recruitment

Participants were identified and recruited by a recruitment agency across four geographically diverse locations in the US (California, Illinois, Maryland, and Missouri). Clinicians confirmed participants’ eligibility (Supplementary Table 7) and provided relevant clinical information. All participants completed an Informed Consent Form prior to any study-related activities. Target sampling quotas ensured a range of demographic and clinical characteristics were represented (e.g., age, type of menopause). A total of 20 interviews, comprised of postmenopausal women (*n* = 10; PM participants) and AET-treated women (*n* = 10; AET participants) experiencing VMS was expected to be sufficient for achieving ‘concept saturation’ [30, 31]. All participants were compensated for participation.

#### Interview procedure

The study was approved and overseen by an Independent Review Board in the US (WCG reference: 20222753). Interviews lasted approximately 75-minutes and were conducted via telephone by trained qualitative interviewers using a semi-structured interview guide.

The CE portion of the interviews started with broad, open-ended questions to facilitate spontaneous elicitation of concepts regarding the experience of VMS. Focused questions were used if concepts of interest had not emerged or been fully explored.

For the CI section, participants were asked to complete the HFDD, PROMIS SD SF 8b, MENQOL, PGI-S and PGI-C items using a ‘think aloud’ approach, sharing their thoughts as they read each instruction/item and selected a response. Participants were asked detailed questions about their interpretation and understanding of instruction/item wording, relevance of concepts, and appropriateness of response options and recall periods. Insights regarding the degree of improvement in HFDD frequency scores that participants would consider meaningful were also obtained.

#### Qualitative analysis

All interviews were audio-recorded and transcribed verbatim, with personally identifiable information redacted. Interview transcripts were analyzed by two researchers trained in qualitative analysis methods using Atlas.Ti [32].

The CE portion of interview transcripts was subject to thematic analysis. Participant quotes pertaining to signs/symptoms, impacts, and coping strategies were assigned corresponding codes in accordance with an agreed coding frame. Codes were applied deductively (based on prior knowledge) and inductively (emerged from the data). Findings were used to assess conceptual coverage of the measures and to refine the draft conceptual model.

Saturation analysis was conducted to determine the appropriateness of the sample size to elicit concepts of interest. Transcripts were chronologically grouped into 4 equal sets (*n* = 5 each) and spontaneously reported sign/symptom and impact concepts identified within each set were iteratively compared. Saturation was deemed achieved if no new symptom/impact concepts were identified in the final interview set [31].

The CI section of interview transcripts was analyzed using codes assigned to each item, instruction, response option, and recall period to indicate whether it was understood and relevant. The level of improvement participants considered meaningful on the HFDD and PGI items was also coded.

## Results

### Qualitative literature review: data sources

The searches returned 1,540 abstracts after duplicates were removed; 211 met the eligibility criteria and were graded (*n* = 20 Grade 1, *n* = 19 Grade 2, *n* = 158 Grade 3, *n* = 14 review articles) by decrease of relevance (Supplementary Tables 3–6). Thirty-nine abstracts (assigned a Grade 1 or 2) were selected for full-text review. Two additional articles identified from a relevant published systematic review were also included. Seventeen records were removed following full-text review due to insufficient data regarding VMS or the target population, or reported the same data as another article. This resulted in a total of 24 sources; 15 described the VMS experience in postmenopausal women [3, 5, 18, 28, 33–43] and nine described the VMS experience in AET-treated women [44–52]. Reviewed studies were mostly conducted with women from the US and Europe; however, studies were also conducted with women from Australia, Brazil, Africa and Asia.

### Qualitative interviews: sample characteristics

Overall, 20 women from the US were interviewed; participants’ demographic and clinical characteristics were diverse (Table 2).

**Table 2:** Overview of participant demographic and clinical characteristics

Characteristics	PM participants (*n* = 10)	AET participants (*n* = 10)	Total (*N* = 20)
**Demographic characteristics**			
**Age**			
Total (mean ± SD)	56.7 ± 6.7	46.2 ± 9.6	51.5 ± 9.7
Min, max	46, 64	26, 58	26, 64
**Ethnicity**,** n (%)**			
Non-Hispanic or Non-Latino	5 (50.0)	8 (80.0)	13 (65.0)
Hispanic or Latino	5 (50.0)	2 (20.0)	7 (35.0)
**Race**,** n (%)**			
White	5 (50.0)	5 (50.0)	10 (50.0)
Black/African American	4 (40.0)	3 (30.0)	7 (35.0)
Other (Hispanic)	1 (10.0)	2 (20.0)	3 (15.0)
**Highest education level**,** n (%)**			
Some high school	3 (30.0)	1 (10.0)	4 (20.0)
High school diploma or General Education Diploma	3 (30.0)	-	3 (15.0)
Some years of college	2 (20.0)	3 (30.0)	5 (25.0)
College or university degree	2 (20.0)	5 (50.0)	7 (35.0)
Graduate or professional degree	-	1 (10.0)	1 (5.0)
**Work status**,** n (%)**			
Working full time	5 (50.0)	4 (40.0)	9 (45.0)
Working part time	-	4 (40.0)	4 (20.0)
Full time homemaker	3 (30.0)	1 (10.0)	4 (20.0)
Not working due to condition	1 (10.0)	-	1 (5.0)
Retired	1 (10.0)	1 (10.0)	2 (10.0)
**Clinical characteristics**			
**Time since menopause reached (last menstrual cycle)**,** n (%)**			
< 2 years	-	2 (20.0)	2 (10.0)
≥ 2–5 years	3 (30.0)	1 (10.0)	4 (20.0)
6–10 years	3 (30.0)	-	3 (15.0)
11 + years	2 (20.0)	-	2 (10.0)
Unknown	2 (20.0)	1 (10.0)	3 (15.0)
**Menopausal status**,** n (%)**			
Premenopausal	N/A	3 (30.0)	3 (15.0)
Perimenopausal	N/A	2 (20.0)	2 (10.0)
Postmenopausal	10 (100)	4 (40.0)	14 (70.0)
Unknown	N/A	1 (10.0)	1 (5.0)
**Reason for reaching menopause**,** n (%)**			
Natural	8 (80.0)	N/A	8 (40.0)
Surgery	2 (20.0)	N/A	2 (10.0)
**Duration experiencing hot flashes**, n (range in years)*			
< 6 years	4 (2.5, 5.3)	8 (0.4, 5.0)	12 (0.4, 5.3)
6–10 years	6 (7.0, 10.0)	1 (N/A)	7 (7.0, 10.0)
> 10 years	-	1 (N/A)	1 (N/A)
**Duration on AET**,** n (%)**			
< 2 years	N/A	5 (50.0)	5 (25.0)
≥ 2–5 years	N/A	5 (50.0)	5 (25.0)
**Current AET treatment**,** n (%)**			
Tamoxifen with/without use of GnRH analogues	N/A	9 (90.0)	9 (45.0)
Aromatase inhibitors with/without use of GnRH analogues	N/A	1 (10.0)	1 (5.0)
Anastrazole	N/A	1 (10.0)	1 (5.0)
**Degree of hot flash interference on sleep over the past 7 days **, n (%)*			
≥ 5 on 0–10 NRS	8 (80.0)	9 (90.0)	17 (85.0)
< 5 on 0–10 NRS	2 (20.0)	1 (10.0)	3 (15.0)
Mean (± SD)	6.1 (± 2.2)	7.4 (± 2.1)	6.8 (± 2.2)
**Degree of hot flash interference on HRQoL over the past 7 days **, n (%)*			
≥ 5 on 0–10 NRS	9 (90.0)	9 (90.0)	18 (90.0)
< 5 on 0–10 NRS	1 (10.0)	1 (10.0)	2 (10.0)
Mean (± SD)	6.4 (± 1.4)	6.8 (± 2.3)	6.6 (± 1.9)

*Participant-reported clinical data at the time of completing demographic form

AET = Adjuvant endocrine therapy, PM = Postmenopausal, NRS = Numeric rating scale, N/A = Not applicable, SD = Standard deviation

### VMS experience findings

**Fig. 1:**
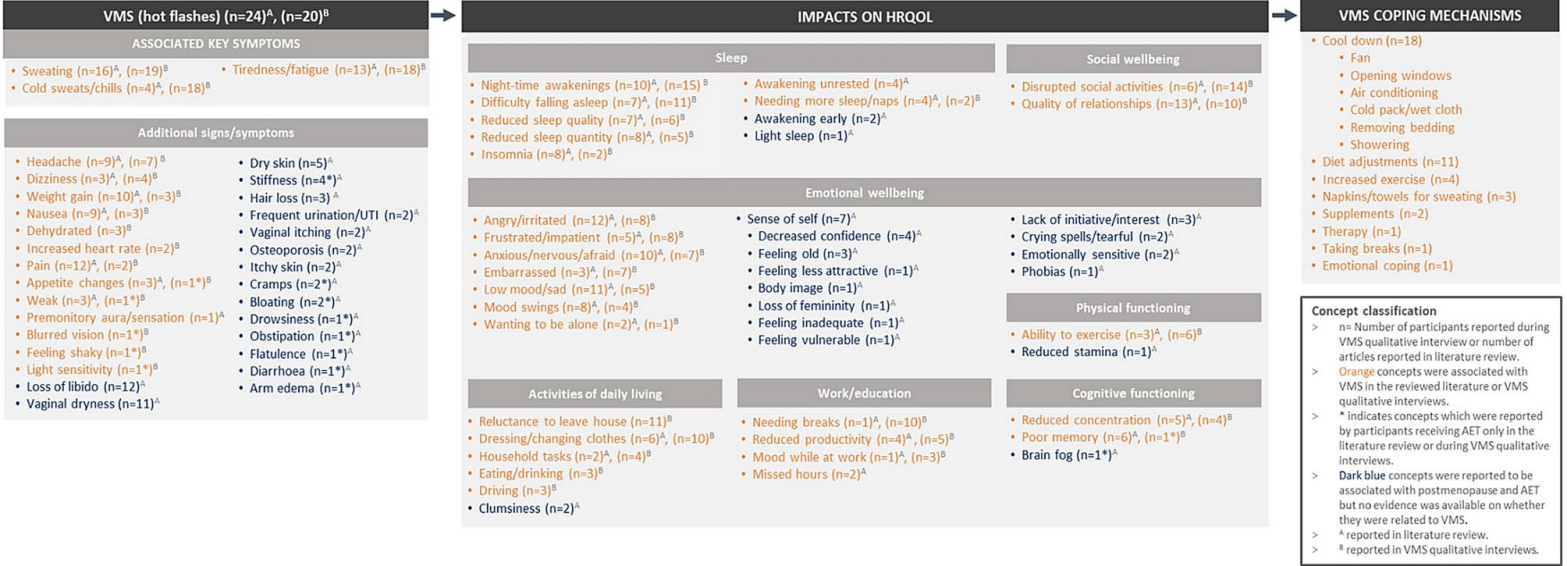
Conceptual model based on literature review and qualitative CE interviews

Findings from the qualitative literature review and CE section of interviews were summarized in a conceptual model, displaying the key signs/symptoms, impacts, and coping mechanisms associated with VMS in postmenopausal and AET-treated women (Fig. 1). Saturation analysis highlighted that no further qualitative CE interviews were necessary since all important signs/symptoms and impacts of VMS had been identified and saturation was achieved (Supplementary Tables 8 and 9).

### Signs/symptoms

Thirty-three signs/symptoms (inclusive of VMS) were identified across the reviewed literature and CE interviews (see Fig. 1 for frequency counts and Table 3 for example quotes). As expected, all sources reported women’s experience of VMS (hot flashes). Interview participants described VMS as heat in several areas of their body (e.g., chest, face and neck) and most commonly reported experiencing hot flashes daily inclusive of night-time (1–7 times per day) and lasting between 2 and 7 min on average.

Three signs/symptoms were categorized as ‘associated key symptoms of VMS (hot flashes)’: sweating, cold sweats/chills, and tiredness/fatigue. These were most commonly identified to be associated with VMS, as exemplified by frequent reports in relation to hot flashes during the CE interviews. Other signs/symptoms associated with VMS mentioned less frequently included headaches, dizziness, weight gain, nausea, dehydration, increased heart rate, and pain. Five signs/symptoms were reported only by AET participants: appetite changes, feeling weak, blurred vision, feeling shaky, and light sensitivity. However, there were no notable differences in sign/symptom reports during the interviews by PM and AET participants.

All interview participants reported using coping mechanisms (in addition to medication) to manage VMS. This included (but was not limited to) methods to keep themselves cool (e.g., using a fan) and adjusting their diet (e.g., drinking more water).

**Table 3:** Overview of VMS and associated key signs/symptoms reported in the literature and by participants during the interviews

Sign/symptom	Literature review (*N* = 24 data sources)	Participant interviews (*N* = 20)	Example supportive quotes
**VMS (hot flashes)**	24/24	20/20	“*[My] body just heats up*,* so my face is hot*,* my neck*,* my chest*,* just my upper body. That’s all the time*,* whether it’s day or night*.” (PM; interview data) “*There’s probably not a day that goes by that I don’t have some type of hot flash… I would probably say that I have more than… five*,* upwards of like maybe seven hot flashes a day.*” (AET; interview data) “*They [hot flashes] get on my nerves. It can be easily seen by other people. Suddenly you become all red.”* (PM; literature review data) [37]
**Sweating**	16/24	19/20	“*The hot flash just comes and you can get just like balls of sweat…you just have to wipe them or get somewhere*,* get something cool*,* a cool rag*.” (PM; interview data) “*I’m usually dripping… I don’t know what’s happening because I’m sleeping*,* so it does wake me up…Sometimes I have to change my clothes because I’m so sweaty and I don’t like how it feels.*” (AET; interview data) *“I sweat a lot and have lots of hot flashes every day*,* but have learnt to tackle them*,* they don’t bother me as much*,* acupuncture did not work for me.”* (AET; literature review data) [44]
**Cold sweats/chills**	4/24	18/20	“*I would say that the chills happen after (a hot flash)… The whole episode lasts maybe an hour and a half so you feel the cold*,* chilly probably in a half hour or so.*” (PM; interview data) “*I do have the cold sweats*,* when I’m having the hot flashes. It comes and it goes. And it’s not consistent to my hot flashes*,* but once in a while.*” (AET; interview data) *“Sometimes I feel internal heat and sometimes cold.”* (PM; literature review data) [43]
**Tiredness/ fatigue**	13/24	18/20	“*Once I’m into my day*,* then the hot flashes can be draining*,* so then it’s like running a marathon… that’s kind of how your body feels from all the sweating and getting hot… By the end of the day*,* I’m exhausted.*” (PM; interview data) “*I don’t know if it’s because I’m so tired from not sleeping or is it just part of a symptom of that? I’m not sure. But yes*,* I do [experience tiredness and fatigue]. Every single day of my life.*” (AET; interview data) *“Wakes up in the middle of the night (around 3 a.m.)*,* sweating. Unable to fall back into sleep. Tired all day.”* (AET; literature review data) [50]

*AET* = Adjuvant Endocrine Therapy treated woman; *PM* = Postmenopausal woman

### Impacts

Seven HRQoL domains were reported to be impacted by VMS during the CE interviews and in the literature: sleep, activities of daily living (ADL), emotional wellbeing, social wellbeing, work/education, physical functioning, and cognitive functioning (see Fig. 1 for frequency counts and Table 4 for example quotes). Interview participants discussed HRQoL impacts spontaneously and the proportion of PM and AET participants reporting on the domains was generally consistent.

VMS was reported to impact sleep, with night-time awakenings most frequently mentioned in the literature and CE interviews. This impact was commonly attributed to feeling hot and experiencing night sweats, with interview participants describing the need to remove bedding during the night. Participants mentioned difficulty falling asleep as well as a reduction in sleep quality and quantity due to VMS, consistent with findings from the literature.

VMS impacted ADL, with interview participants most commonly describing a reluctance to leave the house for fear of experiencing hot flashes in public. Impacts on dressing (e.g., altering the way they dressed) were reported across the CE interviews and literature, and were commonly attributed to feeling too hot or sweating. Interview participants also reported impacts on their ability to carry out chores and on what they could eat or drink (e.g., spicy foods) to avoid triggering hot flashes.

Impacts on emotional wellbeing were frequently reported in the literature and CE interviews. Interview participants described feeling angry/irritated or frustrated/impatient when experiencing a hot flash, or at being unable to control hot flashes. Interview participants discussed feeling anxious/nervous due to not knowing when they will experience a hot flash, why they were experiencing hot flashes, the related physical symptoms (e.g., smell), and the possibility of experiencing hot flashes in public. Feeling embarrassed, particularly in relation to experiencing hot flashes in public, at work, or around friends, was reported. Impacts on work/education, most commonly including taking breaks to cool down or reduced productivity due to hot flashes were also reported. Interview participants reported disruptions to social functioning, such as needing to change where they socialized to avoid places with hotter temperatures. Related to this, interview participants described impacts on the quality of their relationships with partners, family, and friends. Impacts on physical (e.g., difficulty exercising) and cognitive (e.g., difficulty concentrating) functioning were also reported.

**Table 4:** Overview of impact domains and frequently reported concepts reported in the literature and by participants during the interviews

Domain/Concept	Literature review (*N* = 24 data sources)	CE Interview participants (*N* = 20)	Example supportive quotes
**Impacts on sleep**			
Night-time awakenings	10/24	15/20	*“I have strong and frequent hot flashes*,* about once an hour*,* I wake up 4–5 times at night*,* even so*,* the hot flashes are worse during the day.”* (AET; literature review data) [44]
Difficulty falling asleep	7/24	11/20	*“If I’m*,* if I’m hot I [have difficulty falling asleep] because I will pull every piece of—you know*,* I will put on the least I can and pull back the covers or half the time I don’t sleep with covers…”* (PM; interview data)
Reduced sleep quality	7/24	6/20	*“I just have to move and I get hot and I try to uncover and then I try to get comfortable again. And so lately I haven’t been sleeping well.”* (AET; interview data)
Reduced sleep quantity	8/24	5/20	*“What bothers me most today are the hot flashes and the fact that I do not sleep properly anymore… that makes me suffer.”* (PM; literature review data) [28]
**Impacts on ADL**			
Reluctance to leave the house	Not reported	11/20	*“During the summer*,* I just stay inside*,* I like to be in the air conditioning*,* um*,* so it does affect me…Once it gets cooler*,* it will be better*,* but right now since it’s summer*,* I’m limited what I can do… it’s affecting my life.”* (PM; interview data)
Dressing	6/24	10/20	“*These hot flushes*,* they get on my nerves…I’m just about to go out. I’m all dressed up. And suddenly I sweat profusely*,* and I look like a drowned rat. Then I have to change all my clothes.”* (PM; literature review data) [37]
Household tasks	2/24	4/20	*“So if I’m cleaning the house or something…if I get a bad one where I really start to*,* um*,* perspire…I’ll sit down… sometimes turn on a fan and sit there and cool off for a little bit.”* (AET; interview data)
Eating and drinking	Not reported	3/20	*“When I first started getting [hot flashes]… some of my friends said*,* ‘no*,* maybe you need to stop drinking so much coffee’… I tried to*,* to minimize the*,* the caffeine intake.”* (PM; interview data)
Driving	Not reported	3/20	*“I don’t even want to drive to the grocery store.”* (AET; interview data)
**Impacts on emotional wellbeing**			
Angry/irritated	12/24	8/20	*“I get very like irritable too when I’m having a hot flash.”* (PM; interview data)
Frustrated/impatient	5/24	8/20	*“It’s just been very frustrating and it’s something that I just didn’t think I’d be experiencing. Um*,* it’s just*,* it’s awful. It’s awful.”* (AET; interview data)
Anxious/nervous	10/24	7/20	*“Menopause brings anxiety and anguishes*,* especially because of those hot flashes*,* that heat…”* (PM; literature review data) [42]
Embarrassed	3/24	7/20	*“I feel embarrassed because*,* you know*,* I feel like having hot flashes makes people say*,* ‘Oh you’re old*,*’ you know*,* or ‘You’re getting old*,*’ stuff like that… And my family likes to joke around like that a lot like at family gatherings and it is kind of embarrassing.”* (PM; interview data)
Low mood	11/24	5/20	*“I ask myself*,* ‘well*,* are you just using this as an excuse*,* um*,* and you just don’t want to do things’*,* you know what I mean… sometimes it can be*,* um*,* it can be depressing.”* (AET; interview data)
Mood swings	8/24	4/20	*“My husband was asking questions too*,* because of my mood swing. I have a lot of*,* you know*,* mood swings”* (AET; literature review data) [50]
**Impacts on social wellbeing**			
Disrupted social activities	6/24	14/20	*“Uh*,* socially my friends want to go have brunch*,* breakfast*,* lunch*,* and sometimes I say*,* ‘okay*,* let’s go.’ Sometimes I say*,* ‘no. Where? Outside? No. I can’t go outside. It’s too hot. You know*,* inside only*,* only with AC. Cooler is okay.’”* (PM; interview data)
Quality of relationships	13/24	10/20	*“I think it affects sometimes that intimacy if he’s touching me or attempting to… just cuddling*,* nurturing… it affects me because that’s when I don’t want to be touched. I’m too hot.”* (PM; literature review data) [28]
**Impacts on work/education**			
Needing to take breaks	1/24	10/20	*“Well*,* um*,* when I was having them more often*,* they were very bothersome because sometimes I could be at work… sometimes you would just be standing there and you will just start sweating and*,* you know*,* got to go get a drink of water or something.”* (PM; interview data)
Reduced productivity	4/24	5/20	*“I mean there are times where I feel like I’m not doing things as fast as I used to… like I’m not as quick as I used to be.”* (AET; interview data)
**Impacts on physical functioning**			
Ability to exercise	3/24	6/20	*“I don’t really want to participate in like in like weight lifting or*,* um*,* cardio because it’s already uncomfortable. I’m already hot enough.”* (PM; interview data)
**Impacts on cognitive functioning**			
Reduced concentration	5/24	3/20	*“When I*,* when I’m hot*,* when I’m stressed and I’m nervous*,* I can’t*,* I can’t think good. I can’t think good.”* (PM; interview data)
Poor memory	6/24	1/20	*“It’s really funny how things disappear*,* and you forget about a lot of things…”* (AET; literature review data) [49]

*AET* = Adjuvant Endocrine Therapy treated woman; *PM* = Postmenopausal woman

### Conceptual coverage of the PRO measures

The HFDD and MENQOL demonstrated strong conceptual coverage of VMS (hot flashes) and the two key associated signs/symptoms identified in the literature review and CE interviews: sweating and tiredness/fatigue. Additional signs/symptoms (e.g., pain, bloating, dry skin) are also addressed by the MENQOL.

Conceptual coverage of key HRQoL impacts was also demonstrated for the HFDD, PROMIS SD SF 8b, and MENQOL. Most impact concepts identified in the literature and reported by ≥ 50% of interview participants are assessed by these measures, including sleep (e.g., reduced sleep quality), emotional wellbeing (e.g., anger/irritation), social wellbeing (e.g., quality of relationships), work/education (e.g., needing breaks), physical functioning (e.g., ability to exercise), and cognitive functioning (e.g., reduced concentration). Supplementary Table 10 summarizes conceptual coverage of the PRO measures.

### Cognitive interview findings

#### HFDD

Most participants asked (≥ 86.7%) demonstrated an understanding of the instructions for both the Morning and Evening HFDD, and at least 90.0% (*n* ≥ 18/20) of participants demonstrated an understanding of all HFDD items. All participants selected a response option that accurately reflected their experience or reported no concerns when selecting a response option, and most demonstrated an understanding of the recall periods (*n* = 19/20, 95.0%).

Participants confirmed that HFDD items were relevant to their experience (Table 5), with no notable differences between PM and AET participants. The most frequently reported relevant items within the Morning HFDD were ‘number of times woken’, ‘hot flashes experienced’, and ‘hot flash sleep disturbance’ (each *n* = 19/20, 95.0%). At least 85.0% of participants (*n* ≥ 17/20) also reported these items were relevant within the recall period. In general, most participants reported experiencing moderate (*n* = 16/19, 84.2%) or severe (*n* = 15/20, 75.0%) hot flashes during the night and two-thirds (*n* = 13/20, 65.0%) reported experiencing mild hot flashes. When examined within the recall period, slightly fewer participants reported experiencing each hot flash severity type (mild = 45.0%, moderate = 68.4%, severe = 65.0%). Notably, while nearly all PM participants (*n* = 9/10) considered the item assessing ‘mild hot flashes’ relevant, this item was only relevant to 40.0% of AET participants (n = 4/10), as these participants typically experienced more severe hot flashes especially overnight.

All participants asked confirmed the Evening HFDD item assessing ‘hot flashes experienced’ was relevant and all but one participant confirmed this item was relevant within the recall period. Overall, most participants reported experiencing mild (*n* = 15/18, 83.3%) or moderate (*n* = 17/18, 94.4%) hot flashes during the day, but more than half (*n* = 10/18, 55.6%) reported experiencing severe hot flashes. As above, slightly fewer participants reported experiencing each hot flash severity type within the recall period (mild *n* = 12/18, 66.7%; moderate *n* = 15/18, 83.3%; severe *n* = 8/18, 44.4%).

**Table 5:** Summary of relevance of HFDD items

Item, *n* (%) yes	Considered item relevant^a^		Example supportive quotes
	Within recall period	Overall relevance	
**Morning hot flash daily diary**			
Number of times woken	17/20 (85.0)	19/20 (95.0)	“*…at night*,* I wake up a lot*,* so it’s probably like about like four or five times.*” (PM)
Hot flashes experienced	17/20 (85.0)	19/20 (95.0)	“*I had three last night*,* so they woke me up and I had to adjust and get comfortable. So yes*,* I would say three and answer yes to the question.*” (AET)
Number of mild hot flashes	9/20 (45.0)	13/20 (65.0)	*“Total number of mild hot flashes during the night*,* ah*,* sensation of heat without sweating. That would be a 1.*” (PM)
Number of moderate hot flashes	13/19 (68.4)	16/19 (84.2)	“*…yes*,* I went back to sleep*,* so I would say out the eight I would say I had*,* um*,* maybe five that were moderate.*” (AET)
Number of severe hot flashes	13/20 (65.0)	15/20 (75.0)	“*…sensation of heat with sweating*,* causing cessation/stopping of activities. Severe*,* it is very bad*,* two.*” (PM)
Hot flash sleep disturbance	18/20 (90.0)	19/20 (95.0)	“*I would say somewhat because*,* um*,* two of the hot flashes I didn’t have to like actually get out of bed for… And one of them was severe. So for me*,* um*,* I would say somewhat since two were moderate instead of severe.*” (AET)
**Evening Hot Flash Daily Diary**			
Hot flashes experienced	18/19 (94.7)	19/19 (100)	“*It says did you have any hot flashes today since you got up this morning until now? So*,* yes.*” (PM)
Number of mild hot flashes	12/18 (66.7)	15/18 (83.3)	“*It just depends on the day. I could have two or I could have six. It just depends. I probably have more mild than severe is my guess.*” (AET)
Number of moderate hot flashes	15/18 (83.3)	17/18 (94.4)	“*Total*,* um*,* moderate hot flashes during the day of the*,* ah*,* sweating but able to continue activities. One.*” (PM)
Number of severe hot flashes	8/18 (44.4)	10/18 (55.6)	“*I would*,* I would probably—well today*,* um*,* I think I’ve had one.*” (AET)

^a^ Percentages for item relevance are out of the number of participants who were asked

**Fig. 2:**
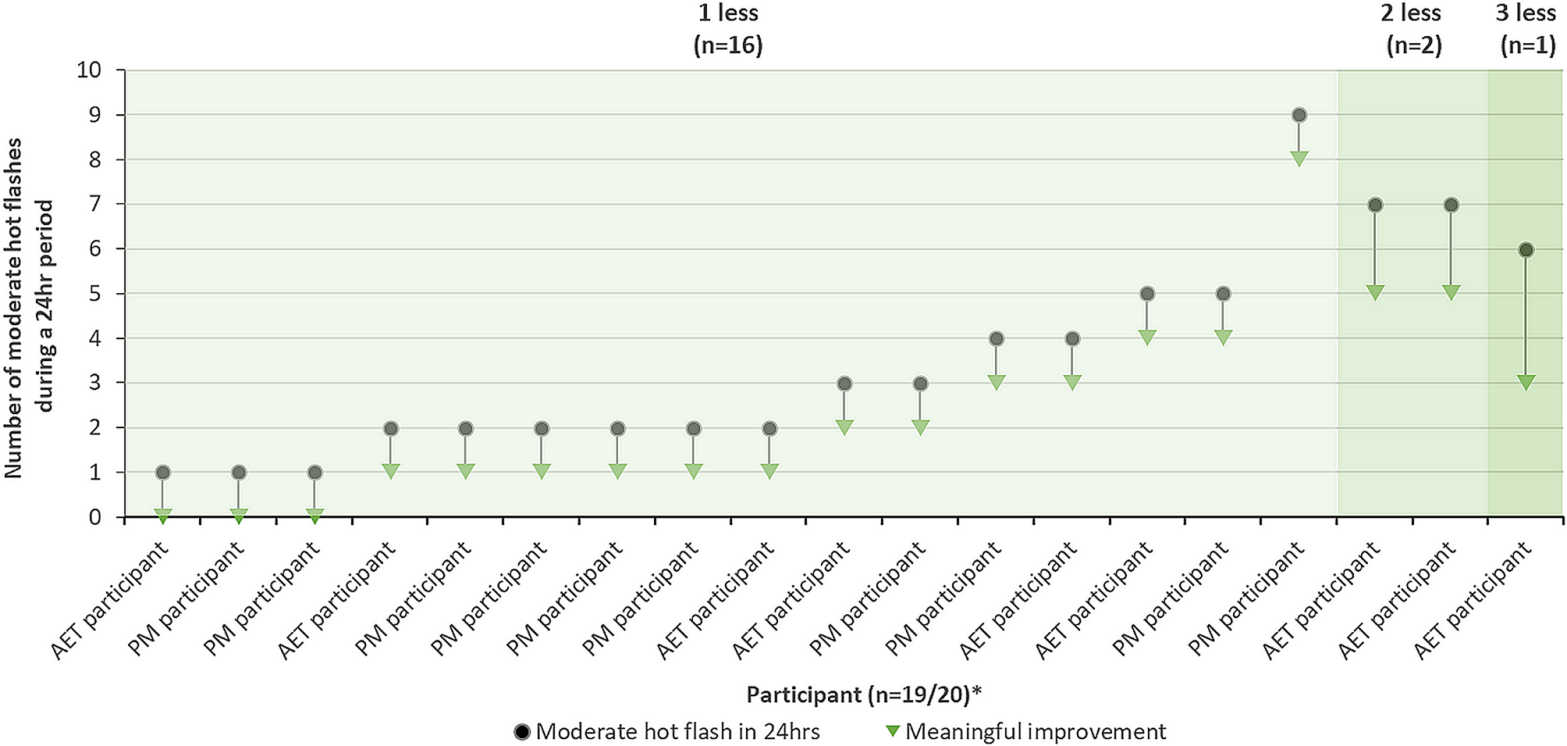
Meaningful change in moderate hot flashes. *Note* *n = 1 reported that a reduction of two moderate hot flashes would be meaningful to her but did not report how many moderate hot flashes this would be a reduced from when thinking about a previous 24-hour period

**Fig. 3:**
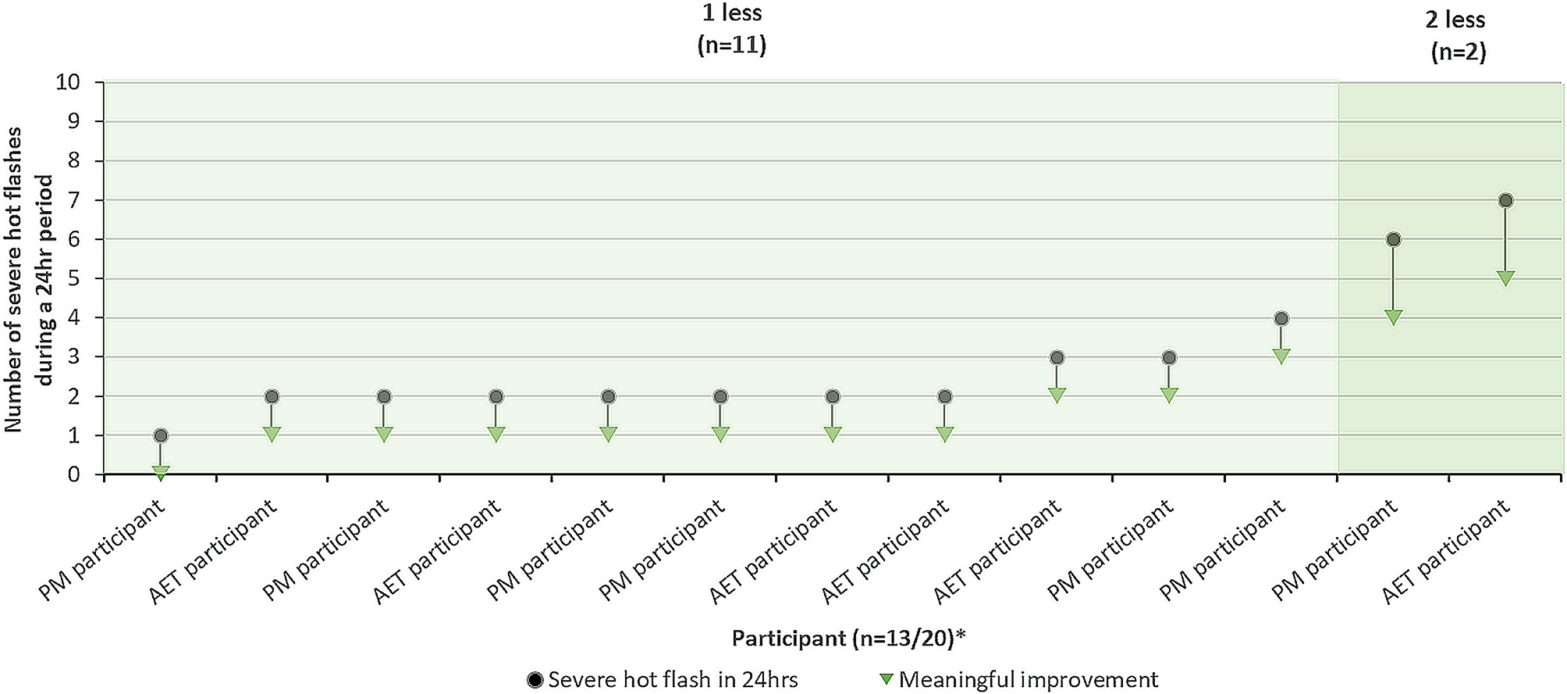
Meaningful change in severe hot flashes. *Note* *n = 6 reported that a reduction of one severe hot flash would be meaningful to them but did not report how many severe hot flashes this would be reduced from when thinking about a previous 24-hour period. The remaining *n* = 1 participant did not provide a response when asked

Responses to HFDD items were used to guide discussions regarding meaningful change. Most participants suggested a reduction of one less moderate hot flash (*n* = 16/20, 80.0%; Fig. 2) and one less severe hot flash (*n* = 17/19, 89.5%; Fig. 3) in 24-hours would be a meaningful improvement. Participants most frequently reported a reduction in moderate and severe hot flashes would lead to improvements in emotional wellbeing (e.g., sense of relief) and sleep (e.g., sleep quality).

### PROMIS SD SF 8b

All participants asked demonstrated an understanding of the PROMIS SD SF 8b items and selected a response option that accurately reflected their experience. Most (*n* = 15/20, 75.0%) also demonstrated an understanding of the recall period.

Participants confirmed that PROMIS SD SF 8b items were relevant to their experience (Table 6), with no notable differences between PM and AET participants. Except for ‘difficulty falling asleep’, all items were relevant to at least 90.0% of participants (*n* ≥ 18/20) within the recall period and in general. While most participants (*n* = 17/20, 85.0%) reported difficulty falling asleep in general, slightly fewer (*n* = 13/20, 65.0%) reported this difficulty within the recall period.

**Table 6:** Summary of relevance of PROMIS SD SF 8b items

Item, *n* (%) yes	Considered item relevant^a^		Example supportive quotes
	Within recall period	Overall relevance	
Item 1: Restless sleep	20/20 (100)	20/20 (100)	*“I had one day in the past seven days*,* one night actually in the past seven days where I woke up. I was really hot. I was sweating.”* (PM)
Item 2: Sleep satisfaction	19/20 (95.0)	19/20 (95.0)	*“It says*,* in the past seven days*,* I was satisfied with my sleep. Again*,* somewhat because I did have to—I did wake up. I did have to move. I did have to adjust myself. And I didn’t get as much sleep as I should have.”* (AET)
Item 3: Refreshing sleep	20/20 (100)	20/20 (100)	*“…not at all… That I woke up refreshed*,* rejuvenated*,* and that hasn’t happened.”* (PM)
Item 4: Difficulty falling asleep	13/20 (65.0)	17/20 (85.0)	*“I’m going to say quite a bit because I*,* I’ve started taking*,* uh*,* melatonin to fall asleep because I—if I don’t*,* I will be up most of the night.”* (AET)
Item 5: Trouble staying asleep	18/20 (90.0)	18/20 (90.0)	*“I would say that’s*,* um*,* that’s always.”***Interviewer**: ***“So that would be***,*** um***,*** sort of every night for you that you experience that?”****“Uh-huh. Yes.”* (PM)
Item 6: Trouble sleeping	19/20 (95.0)	19/20 (95.0)	*“I had trouble sleeping*,* always because of the hot flashes*,* I’ll wake up.”* (AET)
Item 7: Slept enough	20/20 (100)	20/20 (100)	*“Never… Um*,* if I got enough sleep*,* I was able to function I feel like on a—being another space mentally.”* (PM)
Item 8: Sleep quality	20/20 (100)	20/20 (100)	*“My sleep quality was poor. I wouldn’t say very poor because I did get some sleep*,* but I would say poor… I’m waking up*,* I’m turning the fan on*,* turning to off*,* putting the covers on*,* taking them off.”* (AET)

^a^ Percentages for item relevance are out of the number of participants who were asked

### MENQOL

All participants demonstrated an understanding of the MENQOL instructions and most (*n* ≥ 19/20, ≥ 95.0%) demonstrated an understanding of each item. All participants selected a response option that accurately reflected their experience and demonstrated a correct understanding of the recall period in general.

Item relevance varied (Table 7). The most frequently reported relevant items in general were those assessing ‘tiredness’ (*n* = 19/20, 95.0%), ‘lack of energy’ (*n* = 19/20, 95.0%), ‘hot flashes’ (*n* = 18/19, 94.7%), ‘night sweats’ (*n* = 18/20, 90.0%), and ‘sweating’ (*n* = 18/20, 90.0%). Each of these items was also considered relevant within the recall period by at least 85.0% of participants (*n* ≥ 17/20; one participant was not asked about ‘hot flashes’). Items with lower relevance were ‘increased facial hair’ (*n* = 9/20, 45.0%), ‘changes in skin’ (*n* = 9/20, 45.0%), ‘involuntary urination’ (*n* = 8/20, 40.0%), and ‘vaginal dryness’ (n = 9/19, 47.4%), all of which were discussed within the recall period.

Relevance of items was broadly similar across the two subgroups; however, slightly more PM participants considered ‘dissatisfaction with personal life’, ‘decrease in physical strength’, ‘increased facial hair’, ‘changes in skin’ and ‘decrease in sexual desire’ relevant to their experience.

**Table 7:** Summary of relevance of MENQOL items

Item description, *n* (%) yes	Considered item relevant^a^		Example supportive quotes
	Within recall period	Overall relevance	
Item 1 assessed hot flashes	17/19 (89.5)	18/19 (94.7)	*“Yes. I’ve had several. Um*,* today alone*,* I had two and I’m not done yet. I may have another one before I go to bed. That’s usually when it happens. Um*,* neither one of them were bad. You know*,* just*,* you know*,* annoying.”* (AET)
Item 2 assessed night sweats	17/20 (85.0)	18/20 (90.0)	*“I would say a five because*,* um*,* I have been*,* um*,* interrupted in the middle of the night by my night sweats*,* um*,* and they’ve kept me from falling asleep as well as*,* um*,* having difficulty going back to bed.”* (PM)
Item 3 assessed sweating	17/20 (85.0)	18/20 (90.0)	*“I was very bothered. I was uncomfortable*,* uh*,* day and night sweating*,* just miserable. Very aware of it.”* (AET)
Item 4 assessed dissatisfaction with personal life	11/20 (55.0)	11/20 (55.0)	*“Because… it’s*,* um*,* ruining my life. It’s*,* um*,* taking over my life right now.”* (PM)
Item 5 assessed feeling anxious/nervous	15/20 (75.0)	15/20 (75.0)	*“And then this next one*,* feeling anxious or nervous*,* at times*,* but not right now. Um*,* to rate it*,* um*,* when I do get nervous and anxious it’s about a three.”* (AET)
Item 6 assessed poor memory	10/20 (50.0)	10/20 (50.0)	*“… poor memory*,* um*,* I would say yes*,* and I would say 6 because I forget*,* I*,* I get*,* um*,* I feel like I can’t breathe when it gets really bad.”* (PM)
Item 7 assessed accomplishing less	13/20 (65.0)	13/20 (65.0)	*“I feel like I’m not as active as I*,* as I once was. Um*,* accomplishing less than I used to. I would say a three.”* (AET)
Item 8 assessed feeling depressed	11/20 (55.0)	14/20 (70.0)	*“I would say yeah*,* because I*,* I can’t go to a lot of outside functions*,* um*,* or I*,* I just don’t want to be in the heat*,* I don’t want to be with people because I’ll get irritable*,* so yes*,* it’s depressing.”* (PM)
Item 9 assessed being impatient	16/20 (80.0)	16/20 (80.0)	*“Being impatient with other people*,* I would say yes. Um*,* and I would say*,* hmm*,* probably a three on the being impatient with other people.”* (AET)
Item 10 assessed wanting to be alone	11/20 (55.0)	11/20 (55.0)	*“Feelings of wanting to be alone*,* I would say a four on this scale.”* (PM)
Item 11 assessed flatulence	11/20 (55.0)	13/20 (65.0)	*“Next one*,* um*,* wind or gas pains*,* yes*,* sometimes. Uh*,* I would rate it as a three.”* (AET)
Item 12 assessed muscle/joint pain	15/20 (75.0)	15/20 (75.0)	*“Aching in muscles and joints*,* that also happens*,* yes… that will be a number 3.”* (PM)
Item 13 assessed feeling tired	19/20 (95.0)	19/20 (95.0)	*“Feeling tired or worn out*,* I would say yes. And*,* um*,* I would rate that on the zero to six scale with a six being extremely bothered. I would probably rate that as a five.”* (AET)
Item 14 assessed difficulty sleeping	17/20 (85.0)	18/20 (90.0)	*“Difficulty sleeping*,* yes*,* yes. Difficulty sleeping*,* I would say a four.”* (PM)
Item 15 assessed aches in neck/head	14/19 (73.7)	14/19 (73.7)	*“Aches*,* aches in back of neck or head*,* yes. I have been getting that a lot*,* so I’m going to put that at a five.”* (AET)
Item 16 assessed physical strength	14/20 (70.0)	14/20 (70.0)	*“Decrease in physical strength*,* yes… I would say a 6 because I don’t want to move because it would be too hot to move.”* (PM)
Item 17 assessed stamina	14/20 (70.0)	15/20 (75.0)	*“… since I’ve been going through this*,* I mean the medicine has helped*,* but I just definitely feel like I’m just not*,* um*,* I’m just not as active*,* I’m just not as*,* um*,* physically able or*,* ah*,* mentally able as I*,* as I used to be.”* (AET)
Item 18 assessed lack of energy	19/20 (95.0)	19/20 (95.0)	*“… because*,* um*,* I feel like I have no energy.”* (PM)
Item 19 assessed dry skin	11/20 (55.0)	12/20 (60.0)	*“Ah*,* dry skin*,* yes. Um*,* maybe a 3 on dry skin.”* (AET)
Item 20 assessed weight gain	10/20 (50.0)	13/20 (65.0)	*“Weight gain*,* yes. Six*,* very bothered.”* (PM)
Item 21 assessed facial hair	9/20 (45.0)	9/20 (45.0)	*“And increased facial hair*,* oh that’s a good one. You know*,* I would say yes*,* but*,* hmm*,* it’s minimal*,* but there is an increase*,* so on that scale from zero to six*,* I would probably put it as a one.”* (AET)
Item 22 assessed changes in skin	9/20 (45.0)	9/20 (45.0)	*“My skin has changed*,* and it is an annoyance*,* um*,* I am extremely bothered*,* so on a scale of 0 to 6*,* I am extremely bothered by the changes in appearance*,* texture*,* or tone of my skin.”* (PM)
Item 23 assessed feeling bloated	13/20 (65.0)	16/20 (80.0)	*“Feeling bloated*,* um*,* yes. Because I have*,* I have felt some bloat in the past week and I don’t know if it’s dietary related. But the*,* um*,* corresponding scale*,* I would probably list that as a three.”* (AET)
Item 24 assessed low backache	15/20 (75.0)	15/20 (75.0)	*“Low backache*,* yeah. I do have that*,* yes. Oh*,* I’m hitting this too hard. Low backache*,* bothered*,* uh*,* give it a three.”* (PM)
Item 25 assessed frequent urination	12/20 (60.0)	12/20 (60.0)	“*Frequent urination. Yes. Like I said*,* sometimes you got to get up in the middle of the night. I ain’t going to lie. Uh*,* does it bother me? I’m putting it as a four. It’s starting to. It’s not severe*,* but it’s starting to.”* (AET)
Item 26 assessed involuntary urination	8/20 (40.0)	8/20 (40.0)	*“Yes. Involuntary urination when laughing or coughing*,* or coughing*,* that would be a number 2.”* (PM)
Item 27 assessed sexual desire	15/20 (75.0)	15/20 (75.0)	*“Uh*,* decrease in sexual desire*,* I would say*,* uh*,* yes. Three.”* (AET)
Item 28 assessed vaginal dryness	9/19 (47.4)	9/19 (47.4)	*“… I would say*,* vaginal dryness*,* I would say yes*,* and I would say a 6.”* (PM)
Item 29 assessed avoiding intimacy	9/19 (47.4)	12/19 (63.2)	*“Avoiding intimacy*,* yes. Two.”* (AET)

^a^ Percentages for item relevance are out of the number of participants who were asked

### PGI-S and PGI-C items

All participants demonstrated an understanding of the PGI-S items. All participants also confirmed the PGI-S items assessing frequency and severity of hot flashes were relevant to their experience within the recall period and most (*n* = 19/20, 95.0%) confirmed the sleep PGI-S was relevant within this timeframe (though the remaining participant reported experiencing sleep disturbances in general). Most considered a 1-level improvement in the frequency (*n* = 18/19, 94.7%) and severity (*n* = 15/17, 88.2%) of hot flashes to be meaningful, regardless of which response option was originally selected. All participants asked considered a 1-level improvement in sleep problems meaningful.

All participants also demonstrated an appropriate understanding of the PGI-C items. More than half of participants asked reported an improvement of ‘a little better’ or ‘a little less’ was the smallest level of improvement they would consider meaningful for the items assessing severity and frequency of hot flashes (each *n* = 11/19, 57.9%). The remaining participants reported an improvement of ‘much better’ or ‘much less’ was the smallest level they would consider meaningful (n = 8/19, 42.1%). For the sleep PGI-C item, all participants reported ‘a little better’ as the smallest improvement they would consider meaningful.

## Discussion

This qualitative research explored women’s experience of VMS and evaluated content validity of the HFDD, PROMIS SD SF 8b and MENQOL to determine their suitability to assess efficacy in VMS clinical trials. Overall, there were no notable differences in findings between postmenopausal and AET-treated women, suggesting the VMS experience could be similar between these two groups.

### Women’s experience of VMS

Literature review findings provided evidence of the significant disruption caused by VMS on women’s wellbeing, daily life, and work/productivity. Most of the identified literature, however, more broadly focused on the experiences of (post)menopausal [33, 35–37, 39, 43] and AET-treated [47, 49, 50, 52] women, rather than individual signs/symptoms attributed to VMS and their associated impact on HRQoL. This research sought to address this gap by exploring the experience of VMS through conduct of in-depth qualitative interviews.

CE interview results informed refinement of a literature-based conceptual model depicting the experience of VMS. Across both data sources, a total of 33 signs/symptoms and seven impact domains on HRQoL were identified. The qualitative data generated enabled further clarification of which sign/symptom and impact concepts within the model are associated with VMS, across both populations.

All interview participants reported experiences of VMS (hot flashes), consistent with previous literature [37, 40, 41, 43, 46, 48, 49, 52]. Three signs/symptoms (i.e., sweating, cold sweats/chills, and tiredness/fatigue) were identified to be closely related to VMS. While these signs/symptoms were identified as relevant in the literature [3, 18, 28, 44, 52], this study provides further evidence of their relationship to VMS, with ≥ 90.0% of participants reporting these in relation to hot flashes. Other signs/symptoms were identified across participants (headaches, dizziness, weight gain, nausea, dehydration, increased heart rate, and pain); however, these were reported by ≤ 35% of participants and therefore judged to be less relevant for understanding the experience of VMS. Additionally, five signs/symptoms (changes in appetite, light sensitivity, blurred vision, feeling shaky and weak) were reported only by AET participants, with some evidence suggesting these may be a side effect of AET [53].

Participants reported a range of impacts associated with VMS, most notably on sleep (e.g., night-time awakenings), ADL (e.g., dressing), social wellbeing (e.g., social activities), emotional wellbeing (e.g., anger/irritation), and work/education (e.g., needing breaks). While findings are broadly consistent with previous literature [28, 34, 44, 49, 50, 52], interviews identified other ADL impacts not previously reported (e.g., reluctance to leave the house for fear of experiencing hot flashes in public), providing further insights into the VMS experience.

Based on these findings and a review of conceptual coverage, the HFDD, PROMIS SD SF 8b, and MENQOL were identified as PROs suitable for assessment of relevant signs/symptoms and HRQoL impacts in women with VMS associated with menopause and in AET.

### Content validity of the PRO measures

Concepts assessed by the HFDD (VMS frequency and severity), the PROMIS SD SF 8b (sleep disturbances), and the MENQOL questionnaire (menopause-related quality of life) align with those endorsed by women as relevant and important to their experience of VMS during the CE interviews and in the qualitative literature. Although cold sweats/chills were identified as a key symptom associated with VMS (i.e., reported by most interview participants), it was not considered pertinent to measure directly, given women may not consider this as a distinct concept that would need to be reported separately from VMS. This was further supported by the data, where most participants only reported this symptom when probed and only one participant considered it distinct from hot flashes.

Across all measures, participants demonstrated a clear and consistent understanding of the items, instructions, and response options. Items assessed were also considered relevant to participants in general and within the recall periods. Items relevant to less than half of participants were largely those assessed by the MENQOL physical domain (i.e., increased facial hair, changes in skin, involuntary urination, and vaginal dryness). As the MENQOL is intended for use in menopausal women generally, it is plausible that participants in the cognitive interviews emphasized the importance of items more proximal to VMS and reported more distal physical signs/symptoms to be of less importance to them [54]. Further, certain MENQOL items were considered relevant to slightly more PM than AET participants (i.e., dissatisfaction with personal life, decreased physical strength, increased facial hair, changes in skin, and decreased sexual desire). As three AET participants were premenopausal at the time of the interview, and the MENQOL was developed for use in menopausal women, it could be expected that some concepts may be less relevant to these women.

Since completion of this research, content validity of the PROMIS SD SF 8b has been confirmed in a population of postmenopausal women experiencing moderate-to-severe VMS from the US, United Kingdom, and France [28]. Findings of the present study align with that research, providing further evidence to support the relevance of assessing sleep via the PROMIS SD SF 8b in the context of VMS. Most importantly, the qualitative interviews extend evidence of content validity to another population of women with VMS – those treated with AET. To our knowledge, this is the first study which confirms content validity of the PROMIS SD SF 8b in this population.

### Meaningful change in hot flashes and sleep disturbance

The qualitative interviews provide preliminary insights into the level of change in the frequency and severity of hot flashes and the severity of sleep disturbance that women experiencing VMS would consider a meaningful improvement, as assessed by the HFDD and PGI items. Existing research has suggested a reduction of 2.7 to 3.6 moderate-to-severe hot flashes per day as minimal thresholds considered meaningful to women experiencing VMS [55, 56], based on quantitative analysis of clinical trial data. Across both populations, interview participants generally reported a reduction of one moderate hot flash or one severe hot flash in a 24-hour period (7 moderate or severe hot flashes per week) would be meaningful. The few participants who reported a larger reduction (2–3 hot flashes per day or 14–21 hot flashes per week) would be needed, also tended to be those who experienced more hot flashes in a 24-hour period (e.g., 6 or 7 per day). This suggests the number of moderate or severe hot flashes experienced in a day may influence the level of reduction needed for participants to consider the change in frequency meaningful. Additionally, a 1-level improvement in the frequency and severity of hot flashes and severity of sleep disturbance using the PGI items was generally viewed as the smallest level of change participants would consider meaningful. The present qualitative findings supplement subsequent meaningful within-individual change thresholds for the HFDD and PROMIS SD SF 8b scores derived from triangulated estimates using multiple quantitative anchor-based analyses of Phase 3 data [57].

### Strengths and limitations

A strength of this research is the use of comprehensive qualitative methods to explore the experience of VMS in postmenopausal and AET-treated women, including conduct of a targeted qualitative literature review and in-depth qualitative interviews allowing further exploration and consolidation of existing evidence. The interview sample included women with a good representation of demographic and clinical characteristics. Notably, a range of education levels were included, which is important when assessing consistency in understanding of PROs across the target population. Further, the inclusion of women experiencing VMS due to menopause and AET supports the generalizability of the findings to a broader population of women with VMS. Saturation was also achieved, suggesting the sample size was sufficient for a qualitative study.

Nevertheless, findings should be interpreted considering the study limitations. For the literature review, sources were largely limited to specific geographic regions, and often in populations that were not fully representative of the target population (i.e., non-VMS populations). Interview participants were exclusively based in the US, which could impact generalizability of findings to other countries and cultures. To mitigate this, efforts were made to ensure recruitment from geographically diverse locations in the US to maximize cultural and socio-demographic diversity in the sample. Participants also had to be experiencing moderate-to-severe VMS, which could impact the generalizability of the findings to women who only experience mild VMS.

## Conclusion

The results of this qualitative research provide in-depth insights into the experience of VMS in postmenopausal and AET-treated women. The research supported development of a conceptual model and provided evidence to support content validity of the HFDD, the PROMIS SD SF 8b, and the MENQOL questionnaires for use to assess treatment efficacy in VMS clinical trials. Although some evidence exists, research has been completed to substantiate the psychometric validity of the instruments in the target population [57].

## Electronic supplementary material

Below is the link to the electronic supplementary material.

Supplementary Material 1

## Data Availability

The datasets generated and/or analyzed during the current study are not publicly available in order to protect participant confidentiality.

## Abbreviations

ADL: Activities of Daily Living
AET: Adjuvant endocrine therapy
CE: Concept elicitation
CI: Cognitive interview
HFDD: Hot Flash Daily Diary
HRQoL: Health-related quality of life
MENQOL: Menopause-Specific Quality of Life Questionnaire
NK: Neurokinin
PGI-C: Patient-Global Impression of Change
PGI-S: Patient-Global Impression of Severity
PM: Postmenopausal
PRO: Patient-reported outcome
PROMIS SD SF 8b: Patient-Reported Outcomes Measurement Information System Sleep Disturbance Short Form 8b
US: United States
VMS: Vasomotor symptoms
