# Supplementary material for: Qualitative exploration of women’s experiences of vasomotor symptoms to support the content validity of patient-reported outcomes
Source: J Patient Rep Outcomes. 2025 Jul 1;9:79. doi: 10.1186/s41687-025-00914-0 (PMC12214113; doi:10.1186/s41687-025-00914-0)
Supplement: Supplementary file 1 — Supplementary Material 1 [file 41687_2025_914_MOESM1_ESM.docx]

# Supplementary material

| **Supplementary Table 1. Search terms to identify qualitative literature in postmenopause** | | |
| --- | --- | --- |
| **Area of interest** | **Search terms** | **Field** |
| **Condition terms** | exp postmenopause/ OR postmenopausal syndrome/ OR menopause/ | Subject heading^†^ |
|  | postmenopaus* | Keyword |
| **Concepts of interest terms** | exp hot flash/ OR flushing/ OR hot flush/ OR sweating / OR sweat/ OR perspiring/ OR hot temperature/ OR sleep deprivation/ OR sleep latency/ OR sleep quality/ OR sleep disturbance/ OR awake/ OR awakening/ OR menopause symptoms/ OR vasomotor/ OR "quality of life"/ OR daily life activity/ OR "activities of daily living"/ OR daily activities/ OR disability/ OR disabilities/ OR work capacity/ OR wellbeing/ OR well being/ OR mood/ OR psychological OR exp work | Subject heading^†^ |
|  | hot flash* OR flush* OR hot flush* OR body temperature OR facial flush* OR facial red* OR sweat* OR perspir* OR sleep depriv* OR (sleep ADJ1 disturb*) OR sleep latency OR (sleep ADJ2 quality) OR sleep disruption OR awak* OR (menopause ADJ2 symptoms) OR vasomotor OR (quality ADJ2 life) OR QOL OR HRQOL OR burden OR (health ADJ1 status) OR (activit* ADJ3 living) OR ADL OR (daily ADJ2 activit*) OR disab* OR well?being OR mood OR psycholog* OR (work ADJ1 productivity) OR absentee* | Keyword |
| **Methodological terms** | exp qualitative research/ OR qualitative analysis/ OR interviews/ OR interview/ OR interviewers/ OR interviewing/ OR focus groups/ OR phenomenology/ OR ethnography/ OR grounded theory/ OR thematic analysis/ | Subject heading^†^ |
|  | qualitative OR qualitative research OR qualitative analysis OR interview* OR focus group* OR phenomenology OR ethnography OR grounded theory OR thematic analysis OR (concept* ADJ1 elicit*) OR (cognit* ADJ1 interview*) OR (cognit* ADJ1 debrief*) | Keyword |
| *Note: * ¬– An asterisk denotes a search command operator used to retrieve variants of a key word term (e.g., postmenopaus* would retrieve postmenopausal, postmenopause etc.); ? – A question mark denotes a search command operator used to retrieve variants in the spelling of a search term (e.g., well?being would retrieve ‘wellbeing’ and ‘well-being’); “”– Quotation marks denote a search command operator used to retrieve phrases; ADJ – The function ‘ADJ’ denotes a search command operator used to retrieve two search terms that are adjacent (or within a specified number) of each other; / – A forward slash denotes a search command operator used to retrieve a subject heading term; exp – Denotes that the subject heading has been exploded, including all narrower subject headings within its tree hierarchy; ^†^ – Subject heading searches included MeSH terms.* | | |

| **Supplementary Table 2. Search terms to identify qualitative literature in AET-treated women** | | |
| --- | --- | --- |
| **Area of interest** | **Search terms** | **Field** |
| **Condition terms** | exp breast cancer/ OR exp breast neoplasm/ OR breast neoplasm/ OR tamoxifen/ OR exp aromatase inhibitor/ or aromatase inhibitors/ | Subject heading^†^ |
|  | breast cancer OR breast neoplasm* OR tamoxifen OR aromatase inhibitor* OR adjuvant endocrine therap* | Keyword |
| **VMS related terms** | exp hot flash/ OR flushing/ OR hot flush/ OR climacterium/ OR exp menopause/ OR early menopause/ OR postmenopause/ OR sweating / OR sweat/ OR perspiring/ OR hot temperature/ OR menopause symptoms/ OR vasomotor/ | Subject heading^†^ |
|  | (menopaus* ADJ2 symptom*) OR (postmenopaus* ADJ2 symptom*) OR (perimenopaus* ADJ2 symptom*) OR vasomotor OR vasomotor symptom* OR hot flash* OR flush* OR hot flush* OR body temperature OR facial red* OR sweat* OR perspir* | Keyword |
| **Concepts of interest terms** | sleep deprivation/ OR sleep latency/ OR sleep quality/ OR sleep disturbance/ OR awake/ OR awakening/ OR "quality of life"/ OR daily life activity/ OR "activities of daily living"/ OR daily activities/ OR disability/ OR disabilities/ OR work capacity/ OR wellbeing/ OR well being/ OR mood/ OR psychological/ OR exp work/ OR exp job performance/ | Subject heading^†^ |
|  | sleep depriv* OR (sleep ADJ1 disturb*) OR sleep latency OR (sleep ADJ2 quality) OR sleep disruption OR awak* OR (quality ADJ2 life) OR QOL OR HRQOL OR burden OR (health ADJ1 status) OR (activit* ADJ3 living) OR ADL OR (daily ADJ2 activit*) OR disab* OR well?being OR mood OR psycholog* OR (work ADJ1 productivity) | Keyword |
| **Methodological terms** | exp qualitative research/ OR qualitative analysis/ OR interviews/ OR interview/ OR interviewers/ OR interviewing/ OR focus groups/ OR phenomenology/ OR ethnography/ OR grounded theory/ OR thematic analysis/ | Subject heading^†^ |
|  | qualitative OR qualitative research OR qualitative analysis OR interview* OR focus group* OR phenomenology OR ethnography OR grounded theory OR thematic analysis OR (concept* ADJ1 elicit*) OR (cognit* adj1 interview*) OR (cognit* adj1 debrief*) | Keyword |
| *Note: * ¬– An asterisk denotes a search command operator used to retrieve variants of a key word term (e.g.,*  *breast neoplasm* would retrieve breast neoplasm, breast neoplasms etc.); ? – A question mark denotes a search command operator used to retrieve variants in the spelling of a search term (e.g., well?being would retrieve ‘wellbeing’ and ‘well-being’); “”– Quotation marks denote a search command operator used to retrieve phrases; ADJ – The function ‘ADJ’ denotes a search command operator used to retrieve two search terms that are adjacent (or within a specified number) of each other; / – A forward slash denotes a search command operator used to retrieve a subject heading term; exp – Denotes that the subject heading has been exploded, including all narrower subject headings within its tree hierarchy; ^†^ – Subject heading searches included MeSH terms.* | | |

| **Supplementary Table 3. Inclusion/exclusion criteria for qualitative literature screening in postmenopause** | |  |
| --- | --- | --- |
| **Decision** | **Criteria** | |
| **Include** | - Publication type: Journal article (dissertations, books or chapters), conference abstracts and excerpts; AND - Explores the patient experience of the condition in postmenopausal women; AND - Utilises data collected via qualitative methodology; AND - Relevant concept of interest is included in the title and/or abstract (e.g., ‘hot flash’ or ‘vasomotor’). | |
| **Exclude** | - Does not explore postmenopause specifically (e.g., only explores perimenopause); OR - Does not explore the patient experience of the condition; OR - Relevant concept of interest not included (e.g., hot flash, VMS); OR - Linked article is not in the English language; OR - Article does not contain qualitative findings; OR - Article only explores surgical menopause. | |

| **Supplementary Table 4. Abstract grading criteria for postmenopause screening** | |
| --- | --- |
| **Grade** | **Criteria** |
| **1** | - Abstract contains qualitative findings, from the patient perspective - Abstract is focused on postmenopause exclusively - Abstract refers to one of the pre-defined concepts of interest (e.g., hot flash, VMS) |
| **2** | - Abstract is not exclusively based on qualitative research, but does contain findings from the patient perspective - Abstract is not exclusively focused on postmenopause (e.g., includes other menopausal stages) |
| **3** | - Abstract does not contain findings from qualitative methods |
| **Review article** | - Abstract contains qualitative findings, from the patient perspective   *Note: Review articles were not included for data extraction and only used to source additional full-text articles* |

| **Supplementary Table 5. Inclusion/exclusion criteria for qualitative literature screening in AET-treated women** | |  |
| --- | --- | --- |
| **Decision** | **Criteria** | |
| **Include** | - Publication type: Journal article (dissertations, books or chapters), conference abstracts and excerpts; AND - Explores the patient experience of VMS (hot flashes) in women treated with AET; - Utilizes data collected via qualitative methodology; AND - Relevant concept of interest is included in the title and/or abstract (e.g., ‘hot flash’ or ‘vasomotor’). | |
| **Exclude** | - Linked article is not in the English language; OR - Does not explore experience of VMS (hot flashes) specifically (e.g., focuses on breast cancer symptoms/impacts); OR - Does not explore the patient experience of AET treatment or breast cancer from the patient perspective. | |

| **Supplementary Table 6. Abstract grading criteria for AET-treated women screening** | |
| --- | --- |
| **Grade** | **Criteria** |
| **1** | - Abstract contains qualitative findings, from the patient perspective - Abstract refers to one of the identified concepts of interest |
| **2** | - Abstract is not exclusively based on qualitative research, but does contain findings from the patient perspective |
| **3** | - Abstract does not contain findings from qualitative methods |
| **Review article** | - Abstract contains qualitative findings, from the patient perspective   *Note: Review articles were not included for data extraction and only used to source additional full-text articles* |

| **Supplementary Table 7. Overview of interview eligibility criteria** |  |
| --- | --- |
| **Inclusion criteria** | |
| **Postmenopausal women experiencing VMS** | |
| 1. Participant is aged between 40 years to 65 years inclusive and is a postmenopausal female, defined as either:    1. ≥12 months of spontaneous amenorrhea prior to signing of informed consent; or    2. surgical menopause (e.g., due to removal of both ovaries) ≥6 months prior to signing informed consent | |
| 1. Participant is experiencing an average of ≥7 daily hot flashes or ≥50 weekly hot flashes that induce sweating associated with menopause.   **OR**  Participant is receiving treatment for hot flashes but is still experiencing hot flashes that induce sweating, and will have experienced an average of ≥7 daily hot flashes or ≥50 weekly hot flashes with sweating associated with the menopause at any point in the past 2 years. | |
| 1. Participant speaks English and is able to read, write, and fully understand the English language | |
| 1. Participant is willing and able to provide informed consent. | |
| 1. Participant is able to participate in a 75-minute interview, conducted over the telephone or computer/tablet | |
| **Women treated with AET experiencing VMS** | |
| 1. Participant is female aged between 18 to 70 years inclusive, at signing informed consent | |
| 1. Participant is experiencing an average of ≥7 daily hot flashes or ≥50 weekly hot flashes that induce sweating associated with menopause.   **OR**  Participant is receiving treatment for hot flashes but is still experiencing hot flashes that induce sweating, and will have experienced an average of ≥7 daily hot flashes or ≥50 weekly hot flashes with sweating associated with the menopause at any point in the past 2 years. | |
| 1. Participant is receiving AET for breast cancer treatment (e.g., tamoxifen, aromatase inhibitors) for at least 4 weeks prior to signing of informed consent and are expected to still be receiving this at the time of the planned interview. | |
| 1. Participant speaks English and is able to read, write, and fully understand the English language | |
| 1. Participant is willing and able to provide written informed consent | |
| 1. Participant is able to participate in a 75-minute interview, conducted over the telephone or computer/tablet | |
| **Exclusion criteria** | |
| **Postmenopausal women experiencing VMS** | |
| 1. Participant has any active ongoing condition that could cause difficulty in interpreting VMS (e.g., infection that could cause pyrexia, pheochromocytoma, carcinoid syndrome), has any unexplained postmenopausal bleeding or is currently pregnant | |
| 1. Participant has cessation of menses due to contraceptive use, pregnancy/breastfeeding, and/or treatment for a medical condition (e.g., tamoxifen, aromatase inhibitors for breast cancer) | |
| 1. Participant has an uncontrolled psychiatric condition (e.g., schizophrenia, bipolar disorder), or severe physical, neurological or cognitive deficits that might mean they have difficulty understanding the nature, scope, and possible consequences of the study, might influence their responses during the interview or lead them to have difficulty participating in a 75-minute interview | |
| **Women treated with AET experiencing VMS** | |
| 1. Participant has any active ongoing condition that could cause difficulty in interpreting VMS (e.g., infection that could cause pyrexia, pheochromocytoma, carcinoid syndrome), is currently pregnant or less than 3 months since delivery, abortion or stop of lactation prior to signing informed consent | |
| 1. Participant has a diagnosis of metastatic (stage IV) breast cancer, or current or previous history of any malignancy, except for hormone-receptor positive breast cancer (Stage 0-III), basal and squamous cell skin tumors | |
| 1. Participant has had surgery or non-surgical (e.g., chemotherapy, radiotherapy, immunotherapy) treatment for breast cancer within the last 3 months prior to signing informed consent (except use of tamoxifen, aromatase inhibitors, GnRH analogues) | |
| 1. Participant has cessation of menses due to contraceptive use, pregnancy/breastfeeding, and/or treatment/surgery for a medical condition other than breast cancer | |

| **Supplementary Table 8. Saturation analysis of symptoms** | | | | | | | | | | | | | | | | | | | | |
| --- | --- | --- | --- | --- | --- | --- | --- | --- | --- | --- | --- | --- | --- | --- | --- | --- | --- | --- | --- | --- |
| **Symptoms relevant to VMS (hot flashes)** | **Group 1** | | | | | **Group 2** | | | | | **Group 3** | | | | | **Group 4** | | | | |
|  | PT1 | PT2 | PT3 | PT4 | PT5 | PT6 | PT7 | PT8 | PT9 | PT10 | PT11 | PT12 | PT13 | PT14 | PT15 | PT16 | PT17 | PT18 | PT19 | PT20 |
| **VMS (hot flashes) and associated key symptoms** | | | | | | | | | | | | | | | | | | | | |
| Hot flashes | P | P | P | P | P | P | P | P | P | P | P | P | P | P | P | P | P | P | P | P |
| Sweating | P | **S** | P | **S** | **S** | **S** | **S** | **S** | **S** | **S** | **S** | P | **S** | **S** | **S** | **S** |  | **S** | **S** | **S** |
| Cold sweats/chills | P | P | P | P | P | P | P | P | P |  | P | P | P |  | P | P | P | P | **S** | **S** |
| Tiredness/fatigue | **S** | P | P | P |  | P |  | P | **S** | P | **S** | P | P | **S** | P | P | P | P | **S** | **S** |
| **Additional symptoms associated with VMS (hot flashes)** | | | | | | | | | | | | | | | | | | | | |
| Headache |  |  |  | **S** |  | P |  |  |  |  |  |  |  |  | **S** | P |  | P | **S** | **S** |
| Dizziness |  |  | **S** | **S** |  |  |  |  | P |  |  |  |  |  |  |  |  |  | **S** |  |
| Nausea |  |  |  | P |  |  |  |  |  |  |  |  |  |  |  | P |  |  | P |  |
| Weight gain | P |  |  |  |  |  |  |  |  |  |  |  |  | P |  |  |  | P |  |  |
| Dehydration |  | **S** |  |  |  |  |  |  |  |  |  |  | **S** |  |  |  |  |  | **S** |  |
| Pain |  |  |  | P |  |  |  |  |  |  |  |  |  |  |  |  |  | P |  |  |
| Increased heart rate |  |  | **S** |  |  |  |  |  |  |  |  |  |  |  |  | **S** |  |  |  |  |
| Changes in appetite | **S** |  |  |  |  |  |  |  |  |  |  |  |  |  |  |  |  |  |  |  |
| Light sensitivity |  |  |  | **S** |  |  |  |  |  |  |  |  |  |  |  |  |  |  |  |  |
| Blurred vision |  |  |  | **S** |  |  |  |  |  |  |  |  |  |  |  |  |  |  |  |  |
| Feeling shaky | **S** |  |  |  |  |  |  |  |  |  |  |  |  |  |  |  |  |  |  |  |
| Weak |  |  |  |  |  | **S** |  |  |  |  |  |  |  |  |  |  |  |  |  |  |

***S****=First spontaneous report;* ***S****=Spontaneous report; P=Probed report; PT=participant; VMS=vasomotor symptoms.*

| **Supplementary Table 9. Saturation analysis of impact domains** | | | | | | | | | | | | | | | | | | | | |
| --- | --- | --- | --- | --- | --- | --- | --- | --- | --- | --- | --- | --- | --- | --- | --- | --- | --- | --- | --- | --- |
| **Impacts of VMS (hot flashes) on HRQoL** | **Group 1** | | | | | **Group 2** | | | | | **Group 3** | | | | | **Group 4** | | | | |
|  | PT1 | PT2 | PT3 | PT4 | PT5 | PT6 | PT7 | PT8 | PT9 | PT10 | PT11 | PT12 | PT13 | PT14 | PT15 | PT16 | PT17 | PT18 | PT19 | PT20 |
| **Impact domains** | | | | | | | | | | | | | | | | | | | | |
| Activities of daily living | **S** | **S** | **S** | P | P | **S** | **S** | **S** | P | **S** | **S** | P | **S** | **S** | P | **S** | **S** | P | **S** | P |
| Sleep | **S** | **S** | **S** | **S** | **S** | **S** | **S** | **S** | **S** | **S** | **S** | **S** | **S** | **S** | **S** | **S** | P | P | **S** | **S** |
| Emotional wellbeing | **S** | P | **S** | **S** |  | **S** | **S** | **S** | **S** | **S** | **S** | P | **S** | **S** | P | **S** | **S** | **S** | **S** | **S** |
| Social wellbeing | **S** | **S** | P |  |  | **S** |  | **S** | **S** | **S** | **S** | P |  | **S** | **S** | P |  | **S** | **S** | P |
| Work/education | **S** | P |  |  |  |  | **S** | **S** | P | P | P | P | P | **S** | P |  | **S** | P | P | P |
| Physical functioning | **S** | **S** | **S** | **S** |  | **S** |  |  | **S** | **S** |  |  | **S** |  | **S** | **S** |  |  |  | **S** |
| Cognitive functioning | **S** | **S** |  | **S** |  |  |  |  |  |  |  |  |  |  |  | P |  |  |  |  |

*HRQoL=Health-related quality of life;* ***S****=First spontaneous report;* ***S****=Spontaneous report; P=Probed report; PT=participant; VMS=vasomotor symptoms.*

| **Supplementary Table 10. Conceptual coverage of PRO measures** | |
| --- | --- |
| **PRO measure** | **Concepts identified in literature review and/or CE interviews assessed** |
| **Hot Flash Daily Diary (HFDD)** | - **VMS (hot flashes).** - **Associated key symptoms:** Sweating. - **Impacts on HRQoL:** Sleep impacts (e.g., night-time awakenings, reduced sleep quality). |
| **Patient-Reported Outcomes Measurement Information System Sleep Disturbance Short Form 8b (PROMIS SD SF 8b)** | - **Impacts on HRQoL:** Sleep impacts (e.g., night-time awakenings, reduced sleep quality). |
| **Menopause-Specific Quality of Life Questionnaire (MENQOL)** | - **VMS (hot flashes).** - **Associated key symptoms:** Sweating and tiredness/fatigue. - **Additional signs/symptoms:** Pain, headache, weight gain, weak, frequent urination, dry skin, vaginal dryness, loss of libido, flatulence, and bloating. - **Impacts on HRQoL:** Sleep impacts (e.g., night-time awakenings, reduced sleep quality), social wellbeing impacts (e.g., quality of relationships, disrupted social activities), emotional wellbeing impacts (e.g., anxious/nervous, angry/irritated), work/education impacts (e.g., needing breaks, reduced productivity), activities of daily living impacts (e.g., household tasks), physical functioning impacts (e.g., ability to exercise, reduced stamina) and cognitive functioning impacts (e.g., poor memory, reduced concentration). |
